# Evaluation of Xpert^®^ Carba-R Assay Performance from FecalSwab™ Samples

**DOI:** 10.3390/diagnostics15050556

**Published:** 2025-02-25

**Authors:** Nicla Giovacchini, Chiara Chilleri, Ilaria Baccani, Eleonora Riccobono, Gian Maria Rossolini, Alberto Antonelli

**Affiliations:** 1 Department of Experimental and Clinical Medicine, University of Florence, 50134 Florence, Italy; nicla.giovacchini@unifi.it (N.G.); chiara.chilleri@unifi.it (C.C.); ilaria.baccani@unifi.it (I.B.); eleonora.riccobono@unifi.it (E.R.); gianmaria.rossolini@unifi.it (G.M.R.); 2 Microbiology and Virology Unit, Careggi University Hospital, 50134 Florence, Italy; 3 Department of Medical Biotechnologies, University of Siena, 53100 Siena, Italy; 4 NARR Joint Laboratory for Antimicrobial Resistance Research and Control, University of Florence—IRCCS Don Gnocchi Foundation, 50134 Florence, Italy

**Keywords:** clinical microbiology, CPE, CRE, rectal swab, rapid diagnostic, antibiotic resistance, carbapenemases

## Abstract

**Background/Objectives**: The emergence and spread of carbapenemase-producing *Enterobacterales* (CPE) represents a significant challenge prompting the need to optimize diagnostic tools to detect CPE carriers. The Xpert^®^ Carba-R assay (Cepheid, Sunnyvale, CA, USA), a Real-Time PCR-based test, can detect the *bla*_KPC_, *bla*_VIM_, *bla*_OXA-48_, *bla*_NDM_ and *bla*_IMP_ carbapenemase genes directly from rectal swabs. This study assessed the performance of the Xpert^®^ Carba-R assay using FecalSwab™ (Copan, Brescia, Italy), a liquid-based collection device. **Methods**: The first part of the study aimed to establish the FecalSwab^TM^ volume which gave the most similar Ct values to those obtained by the Transystem™ double swab (Copan). The best volume was then used to assess the limit of detection (LoD) for each target and compare the accuracy of different FecalSwab^TM^ storage conditions (room temperature or 4 °C after 16 h compared to T_0_). **Results**: The results indicated that using 200 µL of the FecalSwab™ medium provided reliable Ct values, with the lowest number of invalid samples compared to traditional methods. The average LoDs for different carbapenemases ranged from 4.7 × 10^3^ to 6.8 × 10^3^ CFU/mL. FecalSwab™ showed a better performance after 16 h at room temperature compared to storage at 4 °C. **Conclusions**: This study supports single sampling with the FecalSwab™ medium for both molecular and cultural methods, for the potential optimization of CPE screening.

## 1. Introduction

The emergence and dissemination of carbapenem-resistant *Enterobacterales* (CRE) which occurred in recent years is considered to be one of the most important antibiotic resistance problems. Infections caused by CRE, which typically exhibit a difficult-to-treat resistance (DTR) profile, are associated with a remarkable burden in terms of morbidity, mortality and healthcare-associated expenditures, and are regarded as a major issue of public health interest [1]. Additionally, individuals carrying carbapenemases-producing organisms (CPOs), even without symptoms, can serve as a crucial reservoir for spreading these organisms in the hospital environment, increasing the risk of further infections [2]. Among CPOs, carbapenemase-producing *Enterobacterales* (CPE) hold significant importance in terms of infection control due to the frequent resistance gene dissemination via horizontal gene transfer [3]. Regarding carbapenems resistance, an alarming report has been recently published by the ECDC, especially regarding *Klebsiella pneumoniae*, for which resistance rates are shown to be equal to or above 25% for 30% of the WHO European Region countries [4,5]. Routine clinical practice in numerous regions globally includes the detection of fecal carriers of CPE, as recommended by public health authorities [6,7]. ECDC guidelines stress the importance of accurate and swift identification of colonization by carbapenemase-producing organisms (CPOs), including CPE [8]. Both the CDC and the European Society of Clinical Microbiology and Infectious Diseases (ESCMID) advocate for the periodic screening of hospitalized patients in high-risk units [9]. The early detection of CPE and the implementation of strict infection control measures are essential to control the spread of these multidrug-resistant organisms. Once CPE are detected, active surveillance cultures with stool or rectal swabs should be conducted. Active CRE surveillance testing is an important strategy to control the spread of MDR bacteria, as it allows for the early implementation of timely and effective measures for containment and transmission prevention through contact isolation, positively impacting public health [10].

Nevertheless, such interventions are critically dependent on the accurate and rapid identification of patients colonized by CPOs through laboratory testing. The challenges associated with CRE have compromised our ability to treat common infectious diseases; in this regard, the introduction of rapid and sensitive methodologies for the detection of CPOs is of the utmost importance [11].

There is still no consensus in the literature about the best method to detect CRE colonization, even though rectal swabs have been shown to be a sensitive method with good correlation in the process of screening for active surveillance [12]. Based on that, rectal swabs are considered the main screening method for active surveillance.

In addition, liquid-phase matrix swabs, such as FecalSwab™ (Copan, Brescia, Italy), a modified Cary–Blair medium specifically designed for the recovery, preservation and detection of enteric pathogens, are suitable for the prolonged storage and transport of enteric pathogens and are more appropriate for automated culture than gel swabs [13]. Samples collected with this system are easily processed on automated instruments for both cultural and molecular testing, minimizing manual handling and allowing for fast testing repetition (with the residual liquid volume). Moreover, the use of a single transport medium simplifies the sampling procedures, reducing the healthcare personnel workload, reducing the incidence of sample rejection and limiting patient discomfort, thereby speeding up the diagnostic process. Molecular multiplex-PCR-based assays, which are claimed to overcome the limitations of surveillance culture and conventional molecular tests, have been developed commercially for direct CPO detection from rectal swabs. The primary advantage of these new molecular CPO tests lies in the significantly reduced TAT and increased sensitivity, with a limited necessity for specialized personnel in molecular biology or specific sample manipulation areas, even if at a higher cost [14].

The Xpert^®^ Carba-R (Cepheid, Sunnyvale, CA, USA) is a Real-Time PCR-based assay that allows for the detection of the major carbapenemases (i.e., KPC, NDM, VIM, OXA-48 and IMP) directly from rectal and perirectal swabs, using the random access and fully automated GeneXpert^®^ platform. According to the manufacturer’s recommendations, the Xpert^®^ Carba-R assay should be performed on rectal swabs using the Transystem™ double swab (TDS, Copan).

In this study, we aimed to assess the Xpert^®^ Carba-R assay performance in routine use with FecalSwab™. FecalSwab™ is a liquid-based collection device (Cary–Blair medium) presenting a single flocked swab system, which has been shown to release more organisms than cotton swabs in dry containers and Amies’ rayon- or dacron-based gel swabs.

## 2. Materials and Methods

### 2.1. Study Design

In the first part of the study (2.2), the equivalent amount of FecalSwab™-preserved specimen compared to the TDS unpreserved specimen amount was determined using stool samples spiked in a pool of stool samples with different carbapenemase-producing strains, processed in triplicate with Xpert^®^ Carba-R assay in order to assess the best volume. As the second step (2.3), the limit of detection (LoD) of the Xpert^®^ Carba-R assay was assessed using one representative strain of each carbapenemase and a non-CPE strain as a control. Finally (2.4), the stability of the preserved specimen in FecalSwab™ in different storage conditions and different time slots was examined. The isolates included in the first three steps (producing OXA-48, NDM, KPC, IMP and VIM), were reference strains previously characterized by whole genome sequencing (WGS).

### 2.2. Determination of Amount of FecalSwab™ Medium Required for Equivalent Inoculum

The equivalency of the FecalSwab™-preserved specimens was determined by comparing the cycle threshold (CT) values of unpreserved specimens to those of the various inoculum volumes of preserved specimens.

The stools from healthy volunteers confirmed negative for carbapenemases by Xpert^®^ Carba-R assay were collected, pooled, aliquoted in 900 µL and stored at −80 °C until use. Then, for each strain included in the study, 100 µL of the 0.5 McFarland bacterial suspensions (approximately 1.5 × 10^7^ CFU/mL) prepared in saline solution were added to the negative stool aliquots to obtain spiked samples. Then, TDSs were dipped into the spiked stools and processed following the Xpert^®^ Carba-R assay protocol. In parallel, each FecalSwab™ was dipped into the spiked stools, transferred into their tubes and mixed to disperse fecal material into the medium. Next 200 µL, 400 µL and 800 µL of the FecalSwab™ medium were added to the Cepheid reagent buffer, respectively. Afterwards, 1.7 mL of Cepheid reagent buffer was transferred to the cartridge and processed, in triplicate, following the Xpert^®^ Carba-R Assay protocol.

### 2.3. Analytical Sensitivity of Xpert^®^ Carba-R Assay (LoD Determination)

The limit of detection (LoD) of the Xpert^®^ Carba-R assay was assessed using five strains representative of each carbapenemase [i.e., IT405 *K. pneumoniae* OXA-48 (*n* = 1), CVB-1 *E. coli* NDM-1 (*n* = 1), IT014 *K. pneumoniae* VIM-1 (*n* = 1), IT001 *K. pneumoniae* KPC-3 (*n* = 1) and 16797 *P. aeruginosa* IMP-1 (*n* = 1)]. To determine the analytical sensitivity, 10-fold serial dilutions of a ~0.5 McFarland suspension were prepared to obtain concentrations from ~10^5^ CFU/mL to ~10^1^ CFU/mL. Afterwards, 100 µL of ~10^5^, ~10^4^ and ~10^3^ CFU/mL suspension were added to 900 µL aliquots of CRE-negative stool samples and vortexed thoroughly in order to obtain final concentrations of ~10^4^, ~10^3^ and ~10^2^ CFU/mL, respectively. Viable cell counting was performed by plating 100 µL of ~10^3^, ~10^2^ and ~10^1^ CFU/mL dilutions. Each spiked sample was processed using 200 µL of FecalSwab™ medium. The same experiment was performed with a non-CPE strain as a control. Each experiment was performed in triplicate. *E. coli* ATCC 25922 was used as a negative control strain.

### 2.4. Evaluation of FecalSwab™-Preserved Specimen Stability

To evaluate the capability of the FecalSwab™ medium to preserve nucleic acid in different storage conditions, three FecalSwabs™ were dipped into spiked loose stools with a known concentration of CPE (i.e., 1.5 × 10^7^ CFU/mL), covering the entire swabs. Ten strains representative of each carbapenemase were used to assess the best storage conditions [i.e, IT405 OXA-48-producing *K. pneumoniae* (*n* = 1), ECBZ-1 OXA-48-producing *E. coli* (*n* = 1), CVB-1 NDM-1-producing *E. coli* (*n* = 1), IT001 KPC-3-producing *K. pneumoniae* (*n* = 1), IT201 KPC-2-producing *K. pneumoniae* (*n* = 1), 22-1706 NDM-5-producing *E. coli* (*n* = 1), IT014 and IT062 VIM-1-producing *K. pneumoniae* (*n* = 2) and 16797 and 16714 IMP-1-producing *Pseudomonas aeruginosa* (*n* = 2)]. A volume of 200 µL was transferred in the Xpert^®^ Carba-R cartridge and immediately processed (T0). The remaining volume of the FecalSwab™ medium was stored at room temperature (RT) and at 4 °C, respectively, and processed after 16 h. Subsequently, the mean Ct values obtained from processing the FecalSwab™ by Xpert^®^ Carba-R were calculated compared to those obtained at T0. In this part of the protocol, ten strains representative of each carbapenemase were included. Each experiment was performed in triplicate.

## 3. Results

### 3.1. Evaluation of the Best Volume of the FecalSwab™-Preserved Specimen

In order to determinate the best volume of the FecalSwab™-preserved specimen compared to the TDS, an analysis was carried out using stool samples spiked with ten different carbapenemase-producing strains, respectively [i.e., *E. coli* OXA-48 (*n* = 1), *K. pneumoniae* OXA-48 (*n* = 1), *E. coli* NDM-1 (*n* = 1), *E. coli* NDM-5 (*n* = 1), *K. pneumoniae* KPC-2 (*n* = 1), *K. pneumoniae* KPC-3 (*n* = 1), *K. pneumoniae* VIM-1 (*n* = 2) and *Pseudomonas aeruginosa* IMP-1 (*n* = 2) (Table 1)]. Several invalid results were obtained with 400 µL and 800 µL of the FecalSwab™-preserved specimen (5/30 and 14/30, respectively), and these volumes were excluded from further analysis. On the other hand, no invalid results were obtained when 200 µL of the FecalSwab™-preserved specimen was used, and the assays performed with this volume showed Ct values just slightly higher than those obtained with the TDS, with an average ~ΔCt of 2.57 (range of 0.8 Ct–4.8 Ct). Therefore, a volume of 200 µL was considered to be the amount of FecalSwab™-preserved specimen providing the most similar Ct values compared to the TDS and was selected for the subsequent validation steps.

### 3.2. Analytical Sensitivity of Xpert^®^ Carba-R Assay with FecalSwab Medium (LoD Determination)

In order to determine the LoD of the Xpert^®^ Carba-R assay for each target, five strains representative of each carbapenemase gene were included [i.e., *K. pneumoniae* OXA-48 (*n* = 1), *E. coli* NDM-1 (*n* = 1), *K. pneumoniae* KPC-3 (*n* = 1), *K. pneumoniae* VIM-1 (*n* = 1) and *P. aeruginosa* IMP-1 (*n* = 1)]. The LoD ranged from 5400 to 7100 CFU/mL for the OXA-48-producing *K. pneumoniae*, from 5700 to 8450 CFU/mL for the KPC-3-producing *K. pneumoniae* and from 4150 to 5500 CFU/mL for the NDM-1-producing *E. coli*. The VIM-1- and the IMP-1-producing strains displayed LoD ranges from 440 to 6650 CFU/mL and from 5900 to 6450 CFU/mL, respectively (Table 2). No invalid results were obtained, regardless of the volume of the transport media used, and there was no unspecific amplification of a carbapenemase gene.

### 3.3. Determination of FecalSwab™-Preserved Specimen Stability in Different Storage Conditions

The experiments performed to evaluate the stability of nucleic acid in the FecalSwab™ transport medium at different storage conditions showed that when comparing both room temperature and 4 °C storage after 16 h with T_0_, the former presented lower Ct values for all samples (ΔCt mean of −4.4, range −8.5–+1 Ct) compared to the latter (ΔCt mean of −0.2, range −2.2–+1.5 Ct). No invalid results were obtained (Table 3).

## 4. Discussion

The rapid detection of carbapenemases among CPE in clinical laboratories is crucial to implement the best available therapy and proper infection control measures. Evaluating the implementation of cultural and/or molecular methods should be adapted in each healthcare system depending on the local organization and epidemiology. This study describes the evaluation of the fecal swab as the specimen type for the Xpert^®^ Carba-R assay, a Real-Time PCR test developed to detect the most prevalent and clinically relevant carbapenemase genes, *bla*_KPC_, *bla*_OXA-48_, *bla*_VIM_, *bla*_NDM_ and *bla*_IMP_, directly from rectal swabs. This system, presenting random access and providing rapid results (<1 h), can be placed both in clinical microbiology laboratories as an instrument and used as bedside point of care testing. The latter feature is of particular interest for CRE screening in long-term care structures (including both rehabilitation units and nursing homes), which do not usually present on-site laboratories and outsource microbiology exams. The FecalSwab™ is a rectal swab collection system, currently validated for the cultural screening of CRE [19,20]. The FecalSwab™ processed with the Xpert^®^ Carba-R assay showed the best results using a volume of 200 µL, in agreement with a previous study [21]. The equivalent volume was successively used for the assessment of the Xpert^®^ Carba-R analytical sensitivity to establishing the LoD, which was quite uniform among the different species and resistance genes, even if with a higher LoD compared to the TDS system. Our results align closely with the findings of a previous study, which reported slightly lower or comparable ranges, suggesting that our results are consistent with the established limits of detection for this assay [21]. However, the previous study did not evaluate the best sample storage conditions, and did not use spiked stool samples in the FecalSwab™ medium (only empty FecalSwab™ media) for LoD and best volume evaluation. The LoD differences observed in our study might be due to strain variability (e.g., variation in the copy number of carbapenemase gene-carrying plasmids). In the third part, FecalSwab™ showed an increased sensitivity after 16 h at room temperature compared to storage at 4 °C for the same period, which showed a substantial stability, in agreement with the recommended storage conditions for cultural examination, which guarantee sample stability with a storage at 2–8 °C up to 72 h (FecalSwab™ product insert). To confirm the results of this evaluation, a prospective study should be performed to assess the accuracy and confirm clinical sensitivity and specificity of the Xpert^®^ Carba-R comparing the performance of the Carba-R assay with the cultural method using a collection of clinical FecalSwab^TM^ samples. In conclusion, the use of liquid-based microbiological specimen collection devices offers several advantages for the laboratory workflow, including the possibility of greater standardization and automation to facilitate specimen processing. In contrast, the Transystem™ double swab system, which is not liquid-based, limits automation and is not suitable for multiple media seeding. The main advantage of combining the use of FecalSwab™ is that a single swab can be used for CPE screening by both cultural and molecular methods, as the residual volume can be plated in case of PCR positivity, limiting the number of rectal swabs repetitions. This feature might also be exploited by long-term care laboratories, which would not need screening repetition for the cultural examination of positive cases. Bacterial culture is generally accepted as the gold standard method for the diagnosis of bacterial infectious diseases. The utility of the cultural method depends on the fact that it makes it possible to accurately identify the carbapenemase-producing species and enables the confirmation of the expression of the resistance mechanism detected by the molecular method, allowing antimicrobial susceptibility testing and subsequent antimicrobial stewardship interventions. However, molecular methods and, in particular, rapid/easy to use commercial assays, reduce the time to results, allowing the early application of infection control measures and limiting the possible cost of preventive patient isolation on admission, without a significative increase in laboratory hands-on time and with no requirement for in-batch sample processing [1]. The advantages of molecular methods for weekly CRE carriage monitoring during hospitalization are, on the contrary, less evident and might be replaced by cultural methods to reduce costs. Moreover, two often-overlooked advantages of using a single transport system are reduced patient discomfort and a simpler sample collection process for healthcare providers. In conclusion, using the fecal swab as the specimen type for the Xpert^®^ Carba-R assay provided similar results compared to the validated Transystem™ double swab system, with an optimal 16 h stability at room temperature and acceptable LoD values, and could be considered as an interesting alternative to combine molecular and cultural testing of the same sample.

## Figures and Tables

**Table 1:** Reference carbapenemase-producing strains analyzed for the study and FecalSwab Ct values obtained with a volume of 200 µL in comparison to the TDS Ct values obtained with a volume of 200 µL.

Strain Name	Species	Carbapenem-R Mechanism	TDS (Ct). Mean of Three Experiments	FecalSwab^TM^ (Ct). Mean of Three Experiments	Reference
16797	*Pseudomonas aeruginosa*	IMP-1	23.9	27.2	This Study
16714	*P. aeruginosa*	IMP-1	22.9	27.0	This Study
IT201	*Klebsiella pneumoniae*	KPC-2	23.1	25.9	[15]
IT001	*K. pneumoniae*	KPC-3	23.8	25.5	[15]
CVB-1	*Escherichia coli*	NDM-1	21.2	24.0	[16]
22–1706	*E. coli*	NDM-5	23.0	25.3	[17]
IT405	*K. pneumoniae*	OXA-48	23.6	25.3	[15]
ECBZ-1	*E. coli*	OXA-48	22.3	25.2	[18]
IT014	*K. pneumoniae*	VIM-1	22.1	24.1	[15]
IT062	*K. pneumoniae*	VIM-1	22.3	24.7	[15]

**Table 2:** Limit of detection of the Xpert^®^ Carba-R performed on the FecalSwab™ medium.

Bacterial Species	Target Gene	LoD Average (CFU/mL)	Average Tested Ranges (CFU/mL)
*P. aeruginosa*	IMP-1	6.1 × 10^3^	6.1 × 10^2^–6.1 × 10^4^
*K. pneumoniae*	KPC-3	6.8 × 10^3^	6.8 × 10^2^–6.8 × 10^4^
*E. coli*	NDM-1	4.7 × 10^3^	4.7 × 10^2^–4.7 × 10^4^
*K. pneumoniae*	OXA-48	6.0 × 10^3^	6.0 × 10^2^–6.0 × 10^4^
*K. pneumoniae*	VIM-1	4.8 × 10^3^	2.6 × 10^2^–2.6 × 10^4^

**Table 3:** Evaluation of storage conditions.

Carbapenemase	Replicate	Spiked Sample 1.5 × 10^7^ (Ct)			ΔCt	
		T_0_	Room T (RT, 16 h)	4 °C (16 h)	T_0_-RT	T_0_-4 °C
IMP-1	A	28.7	25.6	29.3	−3.1	−0.6
IMP-1	B	26.5	21.3	26.4	−5.2	0.1
IMP-1	C	27,3	23.6	27.7	−3.7	−0.4
IMP-1	A	28.8	27.4	27.3	−1.4	1.5
IMP-1	B	25.9	26.9	25.6	1	0.3
IMP-1	C	26.4	26.1	26.3	−0.3	0.1
OXA-48	A	24.6	21.5	24.3	−3.1	0.3
OXA-48	B	25.2	21.2	25.6	−4	−0.4
OXA-48	C	23.2	23.1	23.8	−0.1	−0.6
OXA-48	A	24.7	19.4	24.1	−5.3	0.6
OXA-48	B	24.9	18.6	26	−6.3	−1.1
OXA-48	C	24.6	19.8	25.4	−4.8	−0.8
KPC-2	A	26.3	18.9	26.4	−7.4	−0.1
KPC-2	B	26.3	22.2	25.7	−4.1	0.6
KPC-2	C	26.2	20.6	25.3	−5.6	0.9
KPC-3	A	26.1	18.6	26.9	−7.5	−0.8
KPC-3	B	25.3	18.4	24.3	−6.9	1
KPC-3	C	23.3	21.1	25.5	−2.2	−2.2
NDM-5	A	24.6	17.4	24.5	−7.2	0.1
NDM-5	B	27.8	24.6	27.6	−3.2	0.2
NDM-5	C	24.8	20.3	26.2	−4.5	−1.4
NDM-1	A	24.6	20.5	24.9	−4.1	−0.3
NDM-1	B	24.8	19.3	25.2	−5.5	−0.4
NDM-1	C	24.9	23.6	25.5	−1.3	−0.6
VIM-1	A	24.4	17.9	24.8	−6.5	−0.4
VIM-1	B	23.7	20.7	24.7	−3	−1
VIM-1	C	24.1	15.6	24.1	−8.5	0
VIM-1	A	25.5	18.1	25.6	−7.4	−0.1
VIM-1	B	24.4	20.5	25.2	−3.9	−0.8
VIM-1	C	25.4	19	24.6	−6.4	0.8
